# Hippocampal Neurophysiologic Changes after Mild Traumatic Brain Injury and Potential Neuromodulation Treatment Approaches

**DOI:** 10.3389/fnsys.2016.00008

**Published:** 2016-02-09

**Authors:** Fady Girgis, Jonathan Pace, Jennifer Sweet, Jonathan P. Miller

**Affiliations:** Department of Neurosurgery, University Hospitals Case Medical Center, Case Western Reserve UniversityCleveland, OH, USA

**Keywords:** hippocampus, mild traumatic brain injury, neurophysiology, neuromodulation, cognitive dysfunction, drug repositioning

## Abstract

Traumatic brain injury (TBI) is the leading cause of death and disability in individuals below age 45, and five million Americans live with chronic disability as a result. Mild TBI (mTBI), defined as TBI in the absence of major imaging or histopathological defects, is responsible for a majority of cases. Despite the lack of overt morphological defects, victims of mTBI frequently suffer lasting cognitive deficits, memory difficulties, and behavioral disturbances. There is increasing evidence that cognitive and memory dysfunction is related to subtle physiological changes that occur in the hippocampus, and these impact both the phenotype of deficits observed and subsequent recovery. Therapeutic modulation of physiological activity by means of medications commonly used for other indications or brain stimulation may represent novel treatment approaches. This review summarizes the present body of knowledge regarding neurophysiologic changes that occur in the hippocampus after mTBI, as well as potential targets for therapeutic modulation of neurologic activity.

## Introduction

Every year, one out of every 200 people worldwide will suffer a traumatic brain injury (TBI), including 1.7 million people in the United States alone, with an annual associated cost of approximately $76.5 billion (Faul et al., 2010). It is estimated that 5.3 million Americans live with long-term disability as a result of their injury (Centers for Disease Control and Prevention [CDC], 2013). Young men and the elderly are particularly vulnerable because of an increased incidence of trauma and falls, respectively (Susman et al., 2002). TBI is also prevalent in the military, with approximately 20% of all returning deployed American troops suffering some form of brain injury from a blast exposure (Terrio et al., 2009), the vast majority of these being mild injuries.

Mild TBI (mTBI), sometimes termed concussion, accounts for 75% of all head injuries (Faul et al., 2010). Symptoms include headache, sleep impairment, memory deficit, difficulty concentrating (Levin et al., 1987), and an increased incidence of affective disorders such as depression and anxiety (Park et al., 2008). These clinical problems are persistent and severe in 10% of cases, lasting for months to years, sometimes indefinitely (Greig et al., 2014). Due to the striking prevalence and disruptive clinical manifestations of mTBI, research has focused on elucidating the pathophysiological mechanisms underlying primary and secondary brain injury in order to develop therapies that may alter or prevent these processes. More recently, changes in neural circuitry underlying many of the clinical manifestations of mTBI, especially memory and affective disorders, has been studied in an attempt to understand mechanisms of disruption and how modulation of these networks may be used to therapeutic advantage. In order to determine how novel neuromodulation treatments might be able to improve TBI-associated deficits, it is important to establish how individual neurons and subpopulations of neurons contribute to network activity and how these networks respond to injury. This review will discuss neurophysiological changes in the hippocampus associated with mTBI, and ways in which therapeutic neuromodulation might be used to reverse these deficits.

## Biochemical and Genetic Changes in the Hippocampus Following TBI

Neurological injury that occurs after a traumatic insult can result from two different mechanisms: primary and secondary injury. Primary injury refers to the mechanical forces of shearing and compression at the time of impact, while secondary injury involves the subsequent cascade of pathological events that occurs minutes to days after the insult (Greve and Zink, 2009). Such secondary processes include hypoxia/ischemia, edema, raised intracranial pressure, excitotoxity through glutamate release, calcium dysregulation, cytoskeletal proteolysis, free radical oxidative damage, altered synaptic physiology, cytokine release causing inflammation, and ultimately neuronal cell death (Choi et al., 1987; Raghupathi, 2004; Maas et al., 2008; Barkhoudarian et al., 2011; Greig et al., 2014; Merlo et al., 2014). These secondary injury mechanisms are especially important for therapeutic consideration as they can theoretically be altered or inhibited with the aim of preventing injury or protecting brain tissue that has the potential to recover.

There are several commonalities between the biochemical cascades involved in secondary mTBI injury mechanisms and those found in degenerative central nervous system (CNS) diseases such as Parkinson’s disease (PD; Eakin and Miller, 2012; van Bregt et al., 2012), Alzheimer’s disease (AD), Huntington’s disease (HD), and amyotrophic lateral sclerosis (Daneshvar et al., 2011). As a result, pathways that have been extensively characterized in these diseases can be extrapolated and applied to analogous pathways involved in TBI in order to better understand the mechanisms of injury and potential novel therapeutic approaches. This has important implications for diagnosis: a complex mathematical model of semantic predications was recently used to search almost 100,000 citations of neural injury networks and produced a list of 17 potential biomarkers that may indicate mTBI, with glutamate, glucose, and lactate being the most common (Cairelli et al., 2015). In combination with other compounds, such as the apolipoprotein E-4 allele which adversely influences recovery following brain injury (Lawrence et al., 2015), these biomarkers may aid in the early diagnosis of mTBI, allowing the prompt administration of neuroprotective drugs or neuromodulatory therapies.

Glutamate is particularly important, as this excitatory neurotransmitter is known to play a strong role in secondary injury mechanisms throughout the brain. Microdialysis studies, in both rodents (Folkersma et al., 2011) and humans (Chamoun et al., 2010), have shown that glutamate concentrations increase in the acute phase following injury, whereas magnetic resonance spectroscopy (MRS) studies have shown a persistent decrease in glutamate concentrations following injury. The reason for this discrepancy is that microdialysis measures extracellular glutamate, while MRS measures both intra and extracellular glutamate (Guerriero et al., 2015).

Specifically in the hippocampus, glutamate decreases following moderate to severe temporal cortex injury, as measured by *in vivo* proton MRS in a direct cortical injury model in rodents (Harris et al., 2012). Although the hippocampus was considered perilesional tissue in this experiment, the temporal neocortex, i.e., the lesional tissue, also showed a decrease in glutamate in the acute phase. Human MRS studies also in the acute phase following concussion, however, showed no changes in hippocampal glutamate concentrations, despite finding decreased glutamate levels in the motor cortex (Henry et al., 2010). A reason for this discrepancy may be that the human study was in patients with mTBI, whereas the rodent study looked at moderate to severe TBI.

Another important compound altered in the hippocampus after TBI is the neurotransmitter gamma-aminobutyric acid (GABA). The expression of various GABA receptor subunits is changed, causing reductions of inhibitory postsynaptic currents and increases in excitatory post-synaptic currents after TBI in rodents (Hunt et al., 2011; Almeida-Suhett et al., 2015; Drexel et al., 2015). As a result, deficits occur in long-term potentiation, which can translate into cognitive impairment. Similarly, up and down-regulation of other proteins can lead to impairments in hippocampal synaptic transmission, including those involved in the extracellular signal-regulated kinase (ERK) pathways (Atkins et al., 2009), and the soluble N-ethylmaleimide-sensitive factor attachment protein receptor (SNARE) complex (Carlson et al., 2016).

The regulation of gene transcription affects not only the creation of proteins involved in neurotransmission after injury, but also the synthesis of neurons themselves. Because TBI is associated with hippocampal neuronal damage and death, it is important to develop therapeutic strategies to either protect immature neurons or enhance neurogenesis in the hippocampus after injury. Methods shown to drive neurogenesis post injury include forced overexpression of insulin-like growth factor 1 (IGF-1; Carlson et al., 2014), and the transplantation of synthetic human progenitor cells into the hippocampus after TBI (Blaya et al., 2015). Interestingly, TBI severity has been found to correlate with the degree of post-traumatic neurogenesis in rat hippocampus. Whereas mTBI had no effect on neurogenesis, moderate TBI promoted neural stem cell proliferation without increasing neurogenesis, and severe TBI increased neurogenesis (Wang et al., 2015a). A summary of the biochemical and genetic changes following TBI in rodent hippocampus can be found in Table 1.

**Table 1:** **Biochemical changes in rodent hippocampus following TBI, with effects categorized by subsection of the hippocampal complex**.

Structure	Biochemical changes after TBI
CA1	Decreased surface expression of α1, ß2/3, and γ2 subunits of the GABA_A_ receptor caused reductions in the frequency and amplitude of spontaneous and miniature GABA_A_-receptor mediated inhibitory postsynaptic currents after TBI in rats. This led to deficits in long-term potentiation of synaptic transmission (Almeida-Suhett et al., 2015).
CA1, CA3	Expression of GABA_A_ and GABA_B_ receptor subunit mRNAs α4 (GCL, CA3, CA1), α5 (CA1) and γ2 (GCL, CA3, CA1) was up-regulated after TBI in rats, with many of the changes being reversible (Drexel et al., 2015).
CA1, dentate	Mechanical stimulation using a stretchable microelectrode array disrupted bicuculline (GABA_A_ antagonist) induced long-lasting network synchronization 24 h after TBI, despite the continued ability of injured neurons to fire (Kang et al., 2015).
CA4	Action potential and excitatory post-synaptic current frequencies were increased in hilar GABA neurons after TBI in mice, with a further increase observed after photostimulation of dentate granule cell or CA3 pyramidal cell layers (Hunt et al., 2011).
Dentate	TBI severity affected hippocampal neurogenesis in rats: mild TBI did not affect neurogenesis; moderate TBI promoted neural stem cell proliferation without increasing neurogenesis; severe TBI increased neurogenesis (Wang et al., 2015a).
	Survivin (apoptosis protein inhibitor) down-regulation inhibited adult hippocampal neurogenesis, promoted apoptotic cell death, and worsened memory capacity on water maze testing, after TBI in mice (Zhang et al., 2015).
Entire hippocampal complex	Several microRNAs are significantly altered in hippocampal mitochondria and cytoplasm, including elevated levels of miR-155 and miR-223 (play a role in inflammation), after TBI in rats (Wang et al., 2015b).
	Synthetic, human multineurotrophin neural progenitor cells (MNTS1-NPCs) conferred significant preservation of pericontusional host tissues and enhanced hippocampal neurogenesis after TBI in rats (Blaya et al., 2015).
	Inhibition of population spikes was reduced in the Schaffer collateral pathway (CA3 to CA1) 2 days after TBI in rats, while increases in inhibition in the dentate gyrus (corresponding to increased GABA levels) was seen at both 2 and 15 days after injury (Reeves et al., 1997).
	Decreased field excitatory post-synaptic potentials were recorded in hippocampal subfield CA1 in response to electrical stimulation of the Schaffer collaterals, following TBI *in vitro*, indicating that expression of long-term potentiation and synaptic plasticity was inhibited following TBI (Sick et al., 1998).
	Gene transcriptome study identified upregulation of 193 transcripts and downregulation of 21 transcripts in the hippocampus, affecting mostly the transcription of non-neuronal genes, 24 h after mild TBI in mice (Samal et al., 2015).
	Hippocampus nuclear factor of activated T cells (NFAT) c3 levels (expressed in astrocytes) were decreased both in the cytoplasmic and nuclear fractions, while NFATc4 levels (expressed in neurons) were increased in the cytoplasmic fraction but decreased in the nuclear fraction, after TBI in rats (Yan et al., 2014).
	Insulin-like growth factor 1 (IGF-1) promotes neurogenesis after TBI in mice, in that overexpression of IGF-1 resulted in a marked increase in immature neuron density in the subgranular zone at 10 days after injury (Carlson et al., 2014).
	Soluble N-ethylmaleimide-sensitive factor attachment protein receptor (SNARE) complex was reduced in rat hippocampus following TBI, and resulted in a significant reduction in synaptic vesicle number (Carlson et al., 2016).
	TBI in rats results in chronic signaling deficits through the extracellular signal-regulated kinase (ERK)—cAMP response element-binding protein (CREB) pathway in the hippocampus (Atkins et al., 2009).
	Up-regulation of mRNA and protein for Nav1.6 (a voltage-gated sodium channels alpha subunit) occurred in rat hippocampal neurons after TBI (Mao et al., 2010).
	Nuclear factor erythoid related factor 2 (NRF2, transcription factor) mRNA increased significantly post-TBI at 48 and 72 h and 1 week in the hippocampus with a coincident increase in glial fibrillary acidic protein mRNA, overlapping with heme-oxygenase-1, nicotinamide adenine dinucleotide phosphate-quinone-oxidoreductase 1, glutathione reductase, and catalase mRNA (Miller et al., 2014).

*CA, cornu ammonis; GABA, gamma-aminobutyric acid; GCL, granule cell layer; RNA, ribonucleic acid*.

## Hippocampus Biochemical Pathways and Neuroprotective Agents in mTBI

Since mechanisms of secondary injury after mTBI are similar to other diseases, it is likely that drugs that are already in use for other indications may have efficacy for mTBI as well. One example of such a mechanism is the glucagon-like peptide-1 (GLP-1) signaling pathway. GLP-1 was initially described based on its role in the pancreatic regulation of insulin, but receptors are also found in the CNS and changes in this pathway have been implicated in a number of neurodegenerative diseases (Li et al., 2009, 2010, 2012; Martin et al., 2009; Salcedo et al., 2012). Through coupling to a cAMP second messenger pathway, activation of GLP-1 receptors elicits neurotrophic actions such as neuritic growth while protecting neurons against various insults (Perry et al., 2002). As a result, there have been a number of investigations into modulation of this pathway to treat neurological disease.

Exendin-4 is a synthetic GLP-1 receptor agonist currently approved as a drug for the treatment of hyperglycemia in type-2 diabetes that crosses the blood brain barrier (Kastin and Akerstrom, 2003). When administered after mTBI, this neuroprotective agent has been shown to prevent changes in rodent hippocampal gene expression following mTBI. Many of these genes protected by Exendin-4 are also associated with memory loss in AD (which has been associated with TBI), and administration ameliorated memory deficits after injury (Tweedie et al., 2013). Additional rodent studies using markers of cell death have shown that Exedin-4 improved hydrogen peroxide mediated oxidative stress and glutamate toxicity in neuronal cultures *in vitro*. *In vivo*, using markers of cognitive function, the drug ameliorated mTBI-induced deficits in novel object recognition and maze testing, both when administered prior to and after the insult (Eakin et al., 2013; Rachmany et al., 2013). Therefore, data is emerging to support a shift in indication for the use of Exedin-4 from an antihyperglycemic medication to a potential agent to minimize secondary injury after mTBI.

Another agent with potential efficacy for secondary injury after mTBI is N-Acetyl-cystein (NAC), which is currently used as a mucolytic agent and an antidote to acetaminophen overdose. NAC has been shown to have antioxidant and neurovascular-protective effects after mTBI (Ellis et al., 1991; Hicdonmez et al., 2006; Chen et al., 2008) and has been demonstrated to reverse behavioral deficits in mTBI rodents when administered 30–60 min after injury (Eakin et al., 2014). Specifically in the hippocampus, NAC was found to decrease levels of cytosolic-free Ca^2+^ and reactive oxygen species, reduce apoptosis, and lower caspase-3 and -9 neuronal activities, following TBI in rats (Naziroğlu et al., 2014). In humans, NAC has been shown in a randomized double-blind trial to improve auditory, vestibular, and cognitive functional sequelae after blast-induced mTBI in military personnel (Hoffer et al., 2013). Although the mechanism of action in the trauma setting has yet to be elucidated, the 40 year safety profile and ease of administration of the medication makes it an attractive option for widespread use.

Numerous other compounds have been found to exert effects in the hippocampus following injury. As summarized in Table 2, different substances affect different subsections of the hippocampal complex. Although all these investigations were performed in rodents, several of these biochemical compounds may prove to have beneficial effects in humans suffering from cognitive and memory impairments following TBI.

**Table 2:** **Biochemical compounds investigated in rodent hippocampus following TBI, with effects categorized by subsection of the hippocampal complex**.

**Structure**	**Compound**	**Effects**
CA1	*WIN55, 212-2*	WIN55,212-2 (synthetic cannabinoid) restored CA1 interneuron GABAergic signaling after TBI in mice (Johnson et al., 2014).
CA2, CA3	*NT4/5*	Recombinant Neurotrophin-4/5 (Trk-B ligand) increased survival of CA2/3 pyramidal neurons after TBI in rats, but did not improve functional outcome (Royo et al., 2007).
CA1, CA3, Dentate gyrus	*MK-801*	MK-801 (competitive NMDA receptor antagonist) ameliorated hippocampal neuronal loss after TBI in rats, and improved anxiety and hippocampus dependent memory (Sönmez et al., 2015).
Dentate gyrus	*Rapamycin*	Rapamycin (mTOR inhibitor) reduced dentate granule cell area, neurogenesis, and mossy fiber sprouting; increased recurrent excitation of dentate granule cells; and diminished seizure prevalence after TBI in rats (Butler et al., 2015).
	*Ara-C*	Ara-C (arabinofuranosyl cytidine, antimitotic agent) reduced progenitor cell proliferation and neurogenesis in the dentate gyrus, and completely abolished innate cognitive recovery on water maze performance, after TBI in rats (Sun et al., 2015).
	*Clioquinol*	Clioquinol (zinc chelator) reduced progenitor cell proliferation and neurogenesis after TBI in rats (Choi et al., 2014).
Entire hippocampal complex	*DHF*	DHF (7,8-dihydroxyflavone, brain derived neurotrophic factor imitator) increased the number of adult-born immature neurons in the hippocampus, and promoted their dendrite arborization in the injured brain following TBI in mice (Zhao et al., 2015).
	*NAC + selenium*	N-acetylcysteine (NAC) and selenium (antioxidants) decreased levels of cytosolic-free Ca^2+^, apoptosis, cytosolic reactive oxygen species levels, and caspase-3 and -9 activities in hippocampal neurons, after TBI in rats (Naziroğlu et al., 2014).
	*SB-3CT*	SB-3CT (matrix metallopeptidase nine inhibitor) preserved hippocampal neurons and prevented declines in motor function and memory, following TBI in rats Jia et al., 2014).
	*Oxaloacetate + pyruvate*	Oxaloacetate and pyruvate (blood glutamate scavengers) increased hippocampus neuronal survival and neurologic severity scores, after TBI in rats (Zlotnik et al., 2012).
	*Thymoquinone*	Thymoquinone (phytochemical compound) increased hippocampus neuronal densities and malondialdehyde levels, after TBI in rats (Gülsen et al., 2015).
	*Indomethacin*	Indomethacin (anti-inflammatory) suppressed Nogo-A (membrane protein important in axonal remodeling) expression, leading to decreased levels of IL-1β, therefore lessening neuronal damage after TBI in rats (Chao et al., 2012).

*CA, cornu ammonis; GABA, gamma-aminobutyric acid; Trk, tyrosine kinase; NMDA, N-methyl-D-aspartate; mTOR, mammalian Target Of Rapamycin; IL-1β, Interleukin-1 beta*.

## Hippocampal Circuitry and Neurophysiologic Changes After mTBI

In addition to biochemical changes after injury, changes in electrical neural activity also play a role in the sequelae of TBI. The high prevalence of memory deficits after injury point to a relative vulnerability of the hippocampus and mesial temporal structures as compared to the rest of the brain (Hamm et al., 1993; Finset et al., 1999; Comper et al., 2005), and electrophysiologic studies have shown that changes occur in hippocampal circuit excitability after TBI (Witgen et al., 2005; Ortiz and Gutiérrez, 2015). In addition, moderate and severe TBI is associated with neuronal cell loss in several hippocampal regions (Lowenstein et al., 1992) as well as alterations in cellular homeostasis (D’Ambrosio et al., 1999) and dysregulated synaptic transmission (Toth et al., 1997; D’Ambrosio et al., 1998) in this region. On the other hand, mTBI has been associated with more subtle changes in firing patterns that are present even in the absence of morphological changes.

Studies comparing control rats to those who had undergone lateral fluid percussion induced mTBI found changes in working memory in the absence of differences in histology or neuronal loss (Eakin and Miller, 2012). Single unit recordings from cornu ammonis 1 (CA1) and CA3 subfields during a working memory task showed no differences in firing rates or spike characteristics between the two groups, but rats exposed to mTBI were found to have significantly fewer cells with spatiotemporal activity, and this correlated with task performance. Moreover, in a similar experiment looking at the burst characteristics of hippocampal cells during an object exploration task, memory deficits were found to be associated with decreased burst activity of certain subsets of neurons within the pyramidal cell layer (Munyon et al., 2014). Therefore, functional impairment may stem from an alteration in the activity of certain subpopulations of cells within the hippocampus.

These changes in neuronal firing patterns within the hippocampus can persist after TBI and correlate with changes in cognitive function (Witgen et al., 2005). In the analysis of local field potentials after injury, hippocampal broadband power decreased (Paterno et al., 2015), and specifically disruption of theta rhythm (4–7 Hz) has been implicated in the pathophysiology of memory loss (Winson, 1978; Fedor et al., 2010). It has been theorized that using electrical stimulation to artificially counteract and reverse these disrupted firing patterns within the hippocampus and other structures may regulate cellular and network processes and restore functional memory circuits (Diamond et al., 1988; Nakao et al., 2003; Shirvalkar et al., 2010; Tabansky et al., 2014; Hanell et al., 2015). Accordingly, theta burst stimulation (200 Hz in 50 ms trains, five trains per second; 60 mA biphasic pulses) delivered to the fornix of rats with mTBI improved deficits in learning and memory, whereas non-oscillatory low or high frequency stimulation did not (Sweet et al., 2014). In a related manner, theta band stimulation of the medial septal nucleus of mTBI rats resulted in a transient increase in hippocampal theta activity and improved spatial working memory (Lee et al., 2013), whereas 100 Hz gamma stimulation did not (Lee et al., 2015).

In humans, theta burst stimulation of the fornix has been studied in patients undergoing stereo-electroencephalography evaluation for drug-resistant epilepsy, and was shown to produce evoked potentials in the ipsilateral hippocampus. This resulted in significant and reversible improvements in immediate and delayed performance on a visual-spatial memory task (Miller et al., 2015). Temporally patterned stimulation paradigms may therefore present novel neuromodulatory strategies in the treatment of post-traumatic memory loss and cognitive deficits. Potential targets include the hippocampus, its associated white matter tracts, as well as the medial prefrontal cortex. The dysfunction of the latter is thought to play a role in the failure of emotion regulation after mTBI through top down amygdalar regulation (van der Horn et al., 2016), a mechanism that may result in persistent post-concussive symptoms.

## Imaging in mTBI

Recent advances in imaging have allowed for in-depth structural analysis of brain regions both acutely after concussive injury and in the chronic phase. This is important because the ability to identify abnormalities that may previously have been missed is the first step in developing modalities to treat these phenomena, or in the least decide which patients require treatment. Although no single imaging modality has yet proven to be sufficient for all patients with TBI due to the heterogeneity of the condition (Amyot et al., 2015), each method offers its own advantages.

Conventional magnetic resonance imaging (MRI) and computed tomography (CT) are typically negative in mTBI patients with persistent symptoms of post-concussive syndrome. However, compared to controls, mTBI patients were found to have lower cortical thickness of the right temporal lobe and left insula, and increasing number of mTBIs was associated with lower cortical thickness in more brain areas, including bilateral insula, right middle temporal gyrus, and right entorhinal area (List et al., 2015). These findings are worrisome because if further deterioration of these structures occur as part of the aging process, subtle cognitive symptoms may progress to clinical dementia earlier than in age-matched controls. Diffusion tensor imaging is another technique that has recently emerged as a powerful adjunct to conventional MRI. It enables the measurement of fractional anisotropy, a value that indicates the degree of restriction experienced by water molecules diffusing along white matter fibers. Such a tool offers a noninvasive glimpse into the neurophysiology of these tracts and may allow detection of demyelination and axonal damage that would otherwise be missed on a conventional MRI. Indeed, fractional anisotropy was significantly reduced in the right superior longitudinal fasciculus of patients with mTBI and normal MRI as compared with controls, showing that diffusion tensor imaging is more sensitive than standard imaging in this population (Adam et al., 2015). Another study showed similar but subtler findings, as only those with mTBI who had abnormalities on conventional MRI had white matter changes on diffusion tensor imaging (Panenka et al., 2015).

Although increasing abnormality on post-injury CT and MRI correlate with lower neuropsychological performance (Bigler et al., 2015), diffusion tensor imaging has also shown promise in predicting neuropsychological outcomes following mTBI in patients with normal CT and MRI. Immediately following injury and 6 months post-trauma, patients with mTBI showed significant differences in fractional anisotropy and radial diffusivity as compared to controls, specifically in the corona radiata, anterior limb of internal capsule, cingulum, superior longitudinal fasciculus, optic radiation, and genu of corpus callosum, changes which demonstrated associations with neuropsychological outcomes across both time points (Veeramuthu et al., 2015). Another study has shown significantly reduced fractional anisotropy in the internal capsule of both TBI and depression patients (Maller et al., 2010), further underscoring the potential role for diffusion tensor imaging as a noninvasive biomarker useful in the prognostication of post-concussive symptoms.

MRS is another noninvasive technique that measures metabolic changes within brain tissue. In combination with vascular imaging techniques such as transcranial Doppler ultrasound, these modalities have begun to shed light on metabolic processes and the control and regulation of cerebral blood flow following mTBI (Len and Neary, 2011). In the hippocampus, N-acetyl aspartate to choline and N-acetyl aspartate to creatine ratios were decreased in patients with mTBI in comparison to control subjects (Hetherington et al., 2014). Similar changes were found following mTBI in the thalamus, with a reduction of N-acetyl aspartate to creatine and choline to creatine ratios, findings that are classically associated with ischemia (Sours et al., 2015). Of interest, these results correlated with self-reported sensory symptoms, leading the authors to speculate that novel interventions could target these changes in the hopes of treating patients with persistent post-concussive symptoms, as well as use the absence of such imaging features as an indicator to help predict safe return to work.

Magnetoencephalography is an imaging modality largely restricted to the study of epilepsy, but may prove useful as a sensitive detector of local field potentials in brain areas affected by mTBI. A recent innovative study looked at the tracking of eye movements as a marker for sustained attention over time and internal anticipatory control, measurements that are impaired in chronic mTBI while the patients were undergoing magnetoencephalography (Diwakar et al., 2015). Compared to controls, mTBI patients demonstrated impaired eye tracking that was concurrent with abnormal beta activity, which was suppressed in the right parietal cortex and enhanced in the left caudate and frontal-temporal areas. The clinical significance of these findings is yet to be seen, but this is a strong example of novel imaging methods being used in new and original ways to better understand the neurophysiology of injured neural circuits, methods that may alter the manner in which mTBI is diagnosed and treated in the future.

## Future Directions

Direct modulation of neural activity via deep brain stimulation (DBS) currently does not play a direct role in the setting mTBI, but it does carry promise in the treatment of post-concussive affective disorders, trauma-induced tremor, and neurodegenerative disorders. DBS is an effective treatment for certain patients with severe major depression (Lozano et al., 2012) and obsessive-compulsive disorder (Kisely et al., 2014), but has yet to be investigated in the treatment of these disorders following brain injury. Likewise, PD often responds well to DBS of the subthalamic nucleus or globus pallidus internus (Okun, 2012), but data is lacking in the setting of trauma-induced Parkinson’s. However, tremor following severe brain injury has been shown in small studies to respond to both thalamic and pallidal DBS (Issar et al., 2013; Carvalho et al., 2014).

If neurostimulation augments improvements in cognitive deficits after brain injury, memory disorders may present other potential applications for DBS (Shin et al., 2014). Forniceal DBS in a mouse model of Rett syndrome restored *in vivo* hippocampal long-term potentiation and neurogenesis, and rescued contextual fear memory and spatial learning (Hao et al., 2015). In humans, the fornix and nucleus basalis of Meynert are being investigated as possible DBS targets for improvement of memory in the treatment of AD (Sankar et al., 2014; Suthana and Fried, 2014). Although these strategies seem promising, they will likely be tested for efficacy and safety in the setting of severe TBI, prior to being applied in patients with mTBI.

Less invasive neuromodulation options for the treatment of mTBI include transcranial magnetic stimulation (TMS) and transcranial direct current stimulation (TDCS). TMS uses a surface coil to create a low frequency electric current that is delivered to the surface of the brain, while TDCS uses electrodes placed directly on the scalp. There is evidence in severe TBI that TMS may aid in motor recovery, aphasia, visuospatial neglect (Castel-Lacanal et al., 2014), and cognitive function (Polanowska and Seniów, 2010), but high frequencies are contraindicated due to the risk of seizures, especially in the setting of trauma as the injured brain is already predisposed to epileptiform activity. TDCS uses lower currents and therefore has a lower risk of seizures, but subsequently may also have less clinical effect, as current studies show mixed results in the treatment of post-concussive symptoms (Kang et al., 2012; Leśniak et al., 2014; Ulam et al., 2015). TMS is currently the most extensively studied brain stimulation modality in TBI, but most interventions have been non-targeted and focused on the chronic phase of recovery after the injury (Li et al., 2015). As can be expected, better clinical outcomes occur when these neuromodulatory approaches are coupled with cognitive therapy and neurorehabilitation (Chantsoulis et al., 2015; Page et al., 2015).

Finally, nanotechnology and brain-computer interfaces may present new opportunities. By using a computer to bridge the gap between the brain and peripheral nerves in the setting of an injured spinal cord, brain-computer interfaces are “a form of extended embodiment that become integrated into the individual’s conception of himself as an autonomous agent” (Glannon, 2014). Such systems are still in the experimental stage, but offer hope to those patients with severe motor deficits. Similarly, carbon nanotubes are being used to synthesize nanoelectrode arrays that permit real time monitoring and modulation of electrochemical events at the neural and synaptic level (Andrews, 2007), and may offer a glimpse into the future of the field of neuromodulation. As with DBS, however, these modalities will likely be reserved for patients with severe TBI until efficacy, safety, and economic feasibility can be established.

## Conclusion

Despite the high incidence and prevalence of mTBI, only recently have the pathophysiologic mechanisms begun to emerge. Understanding the biochemical signaling pathways involved in the propagation of secondary injury mechanisms has led to the discovery of novel uses for medications currently used for other indications. Biochemical markers and advances in imaging are allowing for earlier diagnosis of concussions, and developments in the area of DBS for the treatment of memory and affective disorders are creating new avenues for the management of post-concussive syndrome. Ultimately, these advances in neuromodulation strive to improve outcomes in mTBI and substantially impact the lives of patients and families suffering from this condition.

## Author Contributions

FG, JP, JS and JPM wrote and edited the manuscript.

## Conflict of Interest Statement

The authors declare that the research was conducted in the absence of any commercial or financial relationships that could be construed as a potential conflict of interest.
